# Factors associated with successful completion of a substance rehabilitation programme at a psychiatric training hospital

**DOI:** 10.4102/sajpsychiatry.v24i0.1280

**Published:** 2018-10-05

**Authors:** Justine Dreyer, Jacobeth M. Pooe, Loveness Dzikiti, Christa Kruger

**Affiliations:** 1 Department of Psychiatry, School of Medicine, University of Pretoria, South Africa; 2 School of Health Systems and Public Health, University of Pretoria, South Africa

## Abstract

**Introduction:**

Comorbid psychiatric and substance use disorders are common globally. Management of either condition is influenced by comprehensive management of the other.

**Aim:**

The aim of this study was to determine which patient and substance factors are associated with completion of a substance rehabilitation programme in psychiatric inpatients.

**Methods:**

The study was conducted at the Substance Rehabilitation Unit (SRU) of Weskoppies Hospital, a psychiatric training hospital in South Africa. It was a hospital-based two-group cross-sectional study comparing clinical files of completers and non-completers of the SRU programme with respect to patient and substance factors.

**Results:**

Most of the patients referred to the SRU were involuntarily admitted, between the ages of 30 and 49 years, male, black or African origin, South African, single, unemployed, never having received a disability grant and with a highest level of education between Grades 8 and 11. Substance-induced disorders, psychotic disorders and Cluster B personality traits were common. Cannabis, alcohol and tobacco were the most frequently used substances. Patients with a lower level of education, who receive a disability grant or who use Nyaope or tobacco, were statistically significantly less likely to complete the SRU programme than those without these factors. Psychiatric diagnosis and general medical comorbidity were not associated with completion.

**Conclusion:**

Completion rates were comparable to those in general substance rehabilitation centres. The association of tobacco smoking and non-completion was in keeping with other research. Low educational level may be a predictive factor of non-completion in this population. This study has yielded several recommendations for substance rehabilitation services in this population and filled a number of research gaps. Further research is still needed, especially with regard to substance rehabilitation in Nyaope-users and the role of disability grants. Creative approaches will be necessary in order to support patients at risk of dropout, in light of resource limitations and the drive towards individualised care.

